# Acute Pancreatitis Leading to the Diagnosis of Presymptomatic Crohn’s Disease: A Pediatric Case Report

**DOI:** 10.7759/cureus.53397

**Published:** 2024-02-01

**Authors:** Yuji Fujita, Keiichi Tominaga, Takanao Tanaka, Akira Yamamiya, Atsushi Irisawa, Kazuyuki Ishida, Takashi Ishige, Shigemi Yoshihara

**Affiliations:** 1 Department of Pediatrics, Dokkyo Medical University, Mibu, JPN; 2 Department of Gastroenterology, Dokkyo Medical University, Mibu, JPN; 3 Department of Diagnostic Pathology, Dokkyo Medical University, Mibu, JPN; 4 Department of Pediatrics, Gunma University Graduate School of Medicine, Maebashi, JPN

**Keywords:** inflammatory bowel diseases, fecal calprotectin, crohn’s disease, anemia, acute pancreatitis

## Abstract

A 14-year-old boy presented with fever and abdominal pain and was diagnosed with acute pancreatitis based on computed tomography findings. The patient had neither diarrhea nor bloody stool but was diagnosed with microcytic anemia. Endoscopic examination revealed a cobblestone pattern and longitudinal ulcer scars in the jejunum. However, no abnormal findings were observed in the ileum or colon. Endoscopic ultrasound-guided fine-needle aspiration was performed from pancreatic body-tail. Pathological examination revealed no evidence of autoimmune pancreatitis (AIP). It was unclear from pathological examination whether idiopathic pancreatitis had self-limitedly improved or whether it was AIP localized to the pancreatic head. The patient was diagnosed with asymptomatic small-bowel Crohn's disease (CD), which may have been two unrelated events of acute pancreatitis. Acute pancreatitis may precede a diagnosis of inflammatory bowel disease. CD with only jejunal involvement (Montreal classification L4) is extremely rare, and we were able to diagnose it early.

## Introduction

Pediatric inflammatory bowel disease (IBD), especially Crohn's disease (CD), is associated with growth failure due to chronic intestinal inflammation, and growth failure may precede gastrointestinal symptoms such as diarrhea and abdominal pain. Thus it is important to diagnose and treat pediatric IBD early. IBD is characterized by various extraintestinal manifestations, such as uveitis, erythema nodosum, primary sclerosing cholangitis, and ankylosing spondylitis. Acute pancreatitis may also occur in IBD patients. In most cases it is caused by medications, such as 5-aminosalicylic acid or azathioprine; however, some present with extraintestinal manifestations such as autoimmune pancreatitis (AIP) [1]. This case report describes a pediatric patient initially presenting with acute pancreatitis. He was subsequently diagnosed with small bowel CD (Montreal classification L4).

## Case presentation

**Figure 1:**
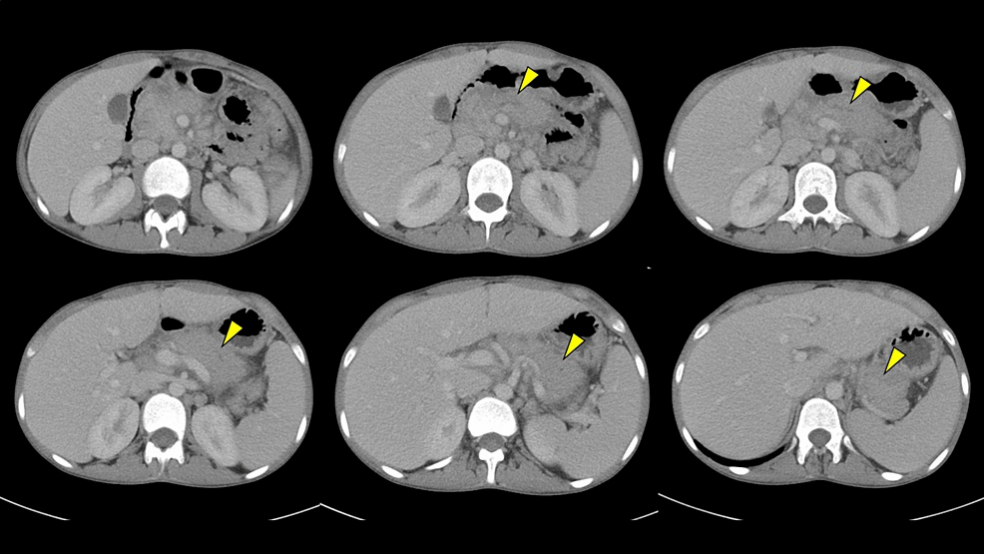
Contrast-enhanced computed tomography scan Contrast-enhanced computed tomography (CT) showed swelling of the pancreas with inflammation spreading to surrounding areas and dilation of the main pancreatic duct in the pancreatic body-tail (yellow arrow).

**Figure 2:**
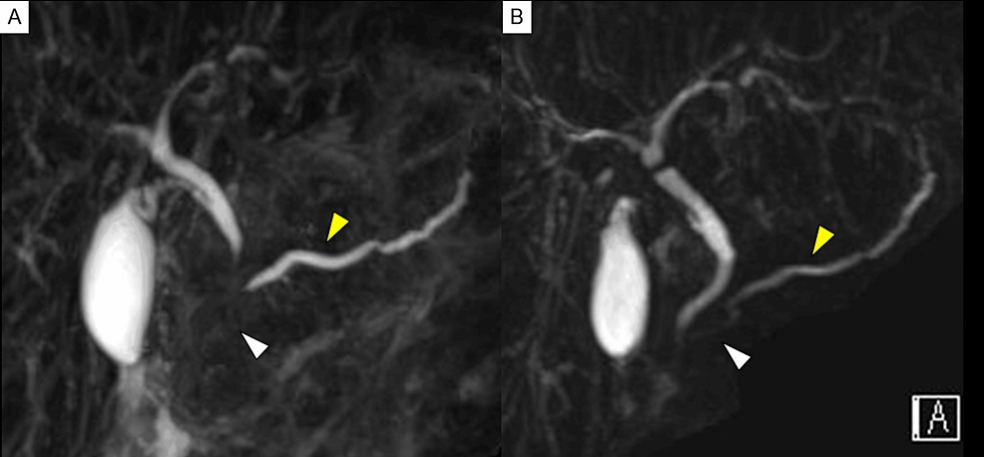
Magnetic resonance cholangiopancreatography Magnetic resonance cholangiopancreatography (MRCP) showed poor visualization of the main pancreatic duct in the pancreatic head with dilatation of the main pancreatic duct in the pancreatic body-tail, and stenotic lower bile duct (yellow arrow) (A: at the time of admission). MRCP showed improvement in pancreatic enlargement, poor visualization of the main pancreatic duct in the pancreatic head, and dilatation of the main pancreatic duct in the pancreatic body-tail (B: one month later).

A 14-year-old boy experienced fever, abdominal pain, and nausea five days ago. The patient had worsened abdominal pain and recurrent vomiting and was brought to our hospital. The patient had a medical history of atopic dermatitis and allergic rhinitis, with no family history of pancreatitis. The patient had no history of insect bites. The patient was also not on any recent medications (including over-the-counter). Physical examination revealed severe epigastric tenderness. The patient had a height of 161.8 cm (-0.3 standard deviation). The patient reported that weight loss (45.5 dropped to 41.4 kg) had occurred in the past two months. Contrast-enhanced computed tomography (CT) scan, revealed swelling of the pancreas with inflammation spreading to surrounding areas, dilation of the main pancreatic duct in the pancreatic body-tail (Figure 1), and no findings of gallstones. The patient was diagnosed with acute pancreatitis. Magnetic resonance cholangiopancreatography (MRCP) also showed poor visualization of the main pancreatic duct in the pancreatic head with dilatation of the main pancreatic duct in the pancreatic body-tail, and stenotic lower bile duct (Figure 2). A blood examination revealed microcytic anemia, hypoalbuminemia, and elevated pancreatic enzyme and C-reactive protein levels (Table 1). No pathogens were noted in the fecal bacterial culture, and the fecal occult blood test result was negative. The fecal calprotectin level was 18,100 mg/kg. The patient’s symptoms and laboratory findings improved with fasting and massive fluid infusion intravenously. The patient was also diagnosed with asymptomatic proximal small bowel CD, which was treated with nutritional therapy.

**Table 1:** Laboratory data of the patient A blood examination revealed microcytic anemia, hypoalbuminemia, elevated pancreatic enzyme, and C-reactive protein levels. WBC: White blood cell; RBC: Red blood cell; MCV: Mean corpuscular volume; PT: Prothrombin time, INR: International normalized time; APTT: Activated partial thromboplastin time; FDP: Fibrinogen/fibrin degradation products; AST: Aspartate aminotransferase; ALT: Alanine aminotransferase; LD: Lactate dehydrogenase; ALP: Alkaline phosphatase; AMY: Amylase; P-AMY: Pancreatic amylase; TG: Triglyceride; BUN: Blood urea nitrogen; UIBC: Unsaturated iron binding capacity; CRP: C-reactive protein; IgG: immunoglobulin G; IgA: immunoglobulin A; IgM: immunoglobulin M; ANA: Antinuclear antibody; MPO: Myeloperoxidase; PR3: Protein 3; ANCA: Antineutrophil cytoplasmic antibody; CMV: cytomegalovirus (normal values)

Hematology			Biochemistry			Immunology		
Complete blood count			AST	12 (10-40)	U/l	IgG	2055 (870-1700)	mg/dl
WBC	16300 (3900-9800)	/µl	ALT	7 (5-40)	U/l	IgG4	150 (11-121)	mg/dl
Neutrophil	73 (40-74)	%	Albumin	2.8 (3.8-5.2)	g/dl	IgE	612.7 (<170)	U/ml
Eosinophil	2.5 (0-6)	%	AMY	230 (37-125)	U/l	ANA	1: 20 (<40)	(-)
Lymphocyte	14.5 (18-59)	%	P-AMY	202 (21-64)	U/l	MPO-ANCA	<1.0 (<3.5)	U/ml
Hemoglobin	10 (13.5-17.6)	g/dl	Lipase	53 (13-55)	U/l	PR3-ANCA	<1.0 (<3.5)	U/ml
MCV	59.9 (82.7-101.6)	fl	TG	63 (50-149)	mg/dl	Infection		
Platelet	70.8 (13.1-36.2)	×10^4^/µl	BUN	7.7 (8-22)	mg/dl	CMV IgM	0.28 (-)	
Coagulation			Creatinine	0.45 (0.61-1.04)	mg/dl	CMV IgG	126 (+)	
APTT	42.3 (24.3-36.0)	sec	Fe	6 (54-200)	µg/dl	Mumps IgM	0.05 (-)	
PT-INR	1.27 (0.85-1.15)		UIBC	196 (104-259)	µg/dl	Mumps IgG	5 (+)	
Fibrinogen	>700 (150-400)	mg/dl	Ferritin	110.2 (39.4-340)	ng/ml	Stool test		
D-dimer	5.8 (<1.0)	µg/dl	CRP	21.77 (<0.14)	mg/dl	Fecal calprotectin	18100 (<50)	mg/kg

**Figure 3:**
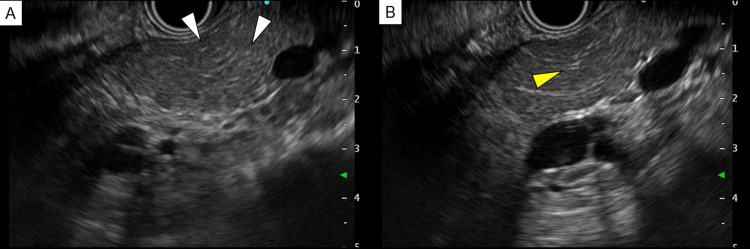
Endoscopic ultrasound-guided fine-needle aspiration Endoscopic ultrasonography-guided fine-needle aspiration showed punctate hyperechoic lesions in the pancreatic parenchyma (A: white arrow) and hyperechoic lesions at the periphery of the main pancreatic duct (B: yellow arrow).

**Figure 4:**
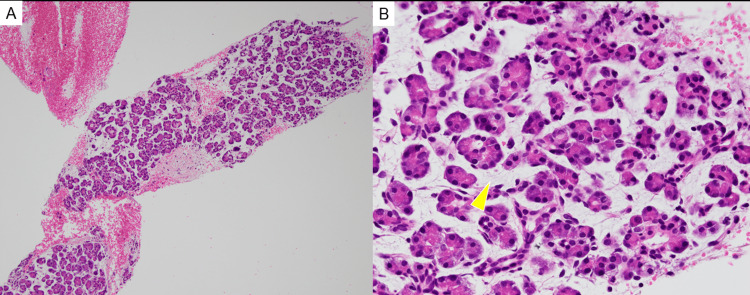
Pathological examination of the body and tail of the pancreas Pathological examination showed that the body and tail of the pancreas were edematous (yellow arrow). However, there was almost no inflammatory cell infiltration, indicating that autoimmune pancreatitis was a less likely diagnosis (original magnification: A × 40; B × 400).

CT showed focal pancreatitis, and following that, IgG4 was found elevated. Therefore endoscopic ultrasound-guided fine-needle aspiration (EUS-FNA) was performed to clarify the pathology. EUS image showed no clear findings of AIP (Figure 3), and histopathological examination showed the absence of inflammatory cell infiltration (Figure 4), indicating that AIP was a less likely diagnosis.

**Figure 5:**
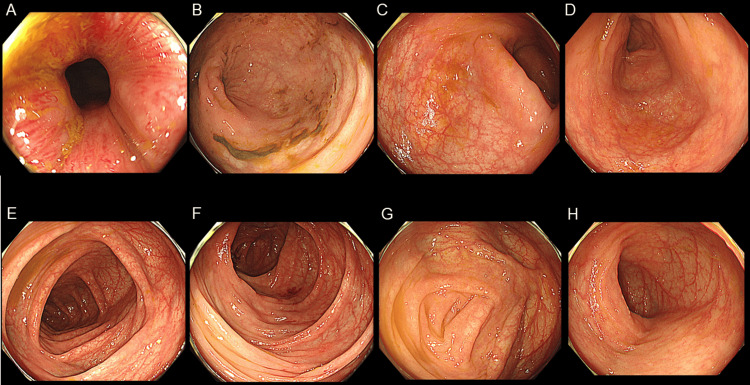
Colonoscopy A colonoscopy revealed no abnormalities in the ileum or colon. A: Anal; B: Rectum; C: Sigmoidal colon; D: Descending colon; E: Transverse colon; F: Ascending colon; G: Cecum; H: Terminal ileum

**Figure 6:**
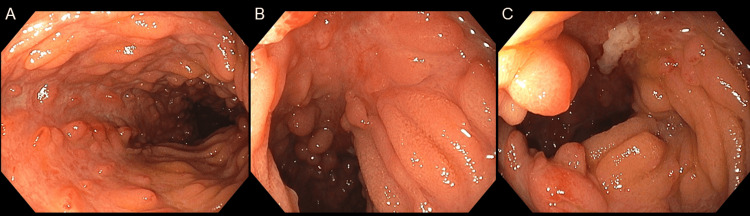
Antegrade double-balloon enteroscopy Antegrade double-balloon enteroscopy showed a cobblestone pattern (A-C) and longitudinal ulcers (C) in the jejunum.

Based on the elevated fecal calprotectin level and chronic microcytic anemia, the complication of IBD was considered. On day 10, a colonoscopy revealed no abnormal findings in the ileum or colon (Figure 5). On day 15, capsule endoscopy showed a cobblestone pattern and longitudinal ulcer scars in the jejunum; however, no abnormal findings were observed in the ileum. On day 17, esophagogastroduodenoscopy revealed a bamboo-joint-like appearance in the stomach and notching of Kerckring’s folds. On day 22, retrograde double-balloon enteroscopy revealed no abnormalities in the ileum or colon. On day 24, antegrade double-balloon enteroscopy revealed a cobblestone-like appearance and longitudinal ulcers (Figure 6).

On day 31, esophagogastroduodenoscopy with a side-view mirror revealed no abnormal morphological findings in the duodenal papilla. On day 37, follow-up MRCP showed imaging improvement of stenosis of the main pancreatic duct in the pancreatic head and of the lower bile duct, as well as improvement of the main pancreatic duct dilatation in the body and tail of the pancreas. This was within a few weeks and without any kind of anti-inflammatory medication, such as corticosteroids. These findings made the diagnosis of idiopathic focal acute pancreatitis of the pancreatic head the most possible.

The patient was also diagnosed with asymptomatic proximal small bowel CD, which was treated with nutritional therapy.

After discharge, the patient experienced bloody stool only once, without other symptoms. Elevated C-reactive protein levels and fecal calprotectin levels were observed. A repeat colonoscopy after four months showed aphthae distally in the terminal ileum and colon, and histopathological examination revealed non-caseating epithelioid cell granulomas. Although he was almost asymptomatic, the patient had extensive small bowel involvement and was therefore started on a biological agent, ustekinumab. Two years have passed since starting ustekinumab and the patient has remained clinically asymptomatic, but the longitudinal ulcer in the jejunum remains and endoscopic remission has not yet been achieved.

## Discussion

The prevalence of pancreatitis among IBD patients is higher than in the general public [2]. Most pancreatitis cases associated with IBD are induced by drugs, specifically azathioprine. Pancreatitis is rarely caused by the spread of CD inflammation to the duodenal papilla or AIP [1]. Moreover, the patient in the present case was not on any recent medications. Additionally, no findings suggested inflammatory spread to the duodenal papilla. Although no pathology findings suggested AIP, the possibility of AIP is not ruled out based on the high serum IgG4 level and pancreatic swelling due to possible sampling error. In AIP, the main pancreatic duct is usually narrowed, but in the patient, the main pancreatic duct in the pancreatic body-tail was dilated. MRCP showed a poorly visualized main pancreatic duct in the pancreatic head, and EUS-FNA was also performed from the pancreatic body and tail, therefore, focal AIP of the pancreatic head is still within the differential diagnoses. MRCP after one month showed improved visualization of the pancreatic duct without any treatment such as corticosteroids, which makes AIP difficult to consider. Thus, in this case, pancreatitis may be considered idiopathic. It is known that idiopathic pancreatitis is associated with IBD and has also been associated with CD [3].

Acute idiopathic pancreatitis affects children more frequently than adults [4,5], and it may precede the diagnosis of IBD. Proximal small bowel CD is rare, frequently asymptomatic, and thus difficult to suspect, similar to the case presented.

In this case, microcytic anemia and elevated fecal calprotectin led to the suspicion that IBD was compatible. Fecal calprotectin is more strongly correlated with small-bowel lesion activity than fecal occult blood [6]. The fecal occult blood test and calprotectin levels were useful diagnostic tests for detecting asymptomatic IBD.

## Conclusions

Our study is significant as we suggest that pediatricians should consider the complications of IBD in patients with acute idiopathic pancreatitis. Pediatricians should keep in mind that there is a rare form of proximal small bowel CD that is frequently asymptomatic. If there is microcytic anemia or elevated fecal calprotectin level, this rare disease type should be taken into consideration and small intestinal lesions should be searched even if there are no abnormalities on CS.
